# Draft Genome Sequence of *Streptomyces fradiae* olg1-1, a Strain Resistant to Nitrone-Oligomycin

**DOI:** 10.1128/genomeA.01252-15

**Published:** 2015-10-22

**Authors:** Aleksey A. Vatlin, Olga B. Bekker, Ludmila N. Lysenkova, Valery N. Danilenko

**Affiliations:** a Vavilov Institute of General Genetics RAS, Moscow, Russia; b Gause Institute of New Antibiotics, Moscow, Russia

## Abstract

We report a draft genome sequence of *Streptomyces fradiae* olg1-1, a mutant strain derived from the model object *S. fradiae* ATCC 19609, which is resistant to nitrone-oligomycin and has a mutation in the DNA-binding domain of a transcriptional regulator PadR.

## GENOME ANNOUNCEMENT

Macrolide antibiotic oligomycin A produces an antibacterial effect on pathogenic actinobacteria, causing actinomycosis, as well as on nonpathogenic strains of *S. fradiae* (1). A series of derivatives of oligomycin A was obtained (1, 2), including nitrone-oligomycin (3), for further research into new anticancer drugs (4) and drugs for the treatment of actinomycosis. There is evidence that the mechanism of action for oligomycin A is based on a chemical interaction with the C-subunit of F_0_F_1_-ATP synthase (5). However, research involving oligomycin A and its derivatives, carried out on the model object *S. fradiae* ATCC 19609, showed that it has additional biotargets (6, 7). The impossibility of obtaining mutants resistant to oligomycin A with a frequency of up to 10^−11^ confirmed the presence of two or more biological targets. The aim of this work was to obtain a mutant strain of *S. fradiae* that is resistant to nitrone-oligomycin and harbors only one resistance mutation.

We carried out whole-genome sequencing of *S. fradiae* olg1-1, a mutant strain resistant to nitrone-oligomycin. The frequency of obtaining mutants was 1 to 2 × 10^9^ CFU. The mutant was selected on solid medium containing 150 µM of nitrone-oligomycin (MIC =10 µM) and phenotypically had Bld-type bad sporulation (8).

Comparative genomic analysis of the mutant strain and the wild-type *S. fradiae* strain ATCC 19609 (9) revealed a mutation in *padR* H(24)R. PadR is a multifunctional transcriptional regulator, which is involved in the control of expression of genes associated with detoxification, virulence, and multidrug resistance in bacteria (10–12). This single nucleotide polymorphism was further confirmed by additional Sanger sequencing. No aerial mycelium developed in the phenotype mutant strain, which suggests that PadR is a transcriptional regulator possibly involved in the process of differentiation, direct resistance to nitrone-oligomycin or negative regulation of genes, and encoding the antibiotic’s biotargets. The inactivation of the *padR* gene may lead to overexpression of these genes, reducing the antibiotic susceptibility.

The genome sequencing of *S. fradiae* strain olg1-1 was carried out using a whole-genome shotgun sequencing approach performed on a Roche 454-GS Junior instrument (Roche, Switzerland). A total of 344,794 reads were generated. All reads were assembled to an initial draft genome of 7,667,096 nucleotides at 21-fold coverage using the GS *de novo* assembler version 3.0 (Roche). The resulting draft genome sequence consists of 208 contigs (193 contigs >500 bp; largest contig, 316,524 bp; overall GC content, 72.8%). The automatic functional annotation results were obtained using the NCBI Prokaryotic Genome Annotation Pipeline (PGAAP, http://www.ncbi.nlm.nih.gov/genome/annotation_prok). The *S. fradiae* olg1-1 genome contains 6,474 predicted genes, 4 rRNA operons, and 65 tRNAs. A total of 5,781 coding sequences (CDSs), 617 pseudogenes, 7 noncoding RNAs (ncRNAs), 4 clustered regularly interspaced short palindromic repeats (CRISPRs), and 77 frameshifted genes were predicted using PGAAP. In addition, 10 insertion sequence (IS) elements were found. The only region of phage activity was identified in the genome sequence (PHAST). This mutation will give a deeper insight to the mechanisms responsible for the resistance of bacteria to nitrone-oligomycin and oligomycin A and can be applied to the development of new antibiotics with a novel mechanism of action.

### Nucleotide sequence accession numbers.

This whole-genome shotgun project has been deposited at DDBJ/EMBL/GenBank under the accession number LGSP00000000. The version described in this paper is the first version, LGSP01000001.
